# Designed Ankyrin Repeat Proteins: A New Class of Viral Entry Inhibitors

**DOI:** 10.3390/v14102242

**Published:** 2022-10-12

**Authors:** Marcel Walser, Jennifer Mayor, Sylvia Rothenberger

**Affiliations:** 1 Molecular Partners AG, Wagistrasse 14, 8952 Zurich-Schlieren, Switzerland; 2 Spiez Laboratory, Federal Office for Civil Protection, Austrasse, 3700 Spiez, Switzerland; 3 Institute of Microbiology, University Hospital Center and University of Lausanne, Rue du Bugnon 48, 1011 Lausanne, Switzerland

**Keywords:** ankyrin repeat protein, protein scaffold, protein design, DARPin, antiviral therapy, multispecificity, emerging virus, SARS-CoV-2, COVID-19

## Abstract

Designed ankyrin repeat proteins (DARPins) are engineered proteins comprising consensus designed ankyrin repeats as scaffold. Tightly packed repeats form a continuous hydrophobic core and a large groove-like solvent-accessible surface that creates a binding surface. DARPin domains recognizing a target of interest with high specificity and affinity can be generated using a synthetic combinatorial library and in vitro selection methods. They can be linked together in a single molecule to build multispecific and multifunctional proteins without affecting expression or function. The modular architecture of DARPins offers unprecedented possibilities of design and opens avenues for innovative antiviral strategies.

## 1. Introduction

The emergence of new viruses and their continuous spread into the human population emphasizes the need for innovative therapeutics to fight viral infections. Currently, among the U.S. Food and Drug Administration (FDA)-approved antiviral compounds, the majority are small molecules, whereas a few are larger entities, such as proteins (monoclonal antibodies and interferons), peptides, and oligonucleotides [1,2]. The emergence of severe acute respiratory syndrome coronavirus 2 (SARS-CoV-2) and the pandemic highlighted the importance of neutralizing monoclonal antibodies (mAbs) to fight coronavirus disease 2019 (COVID-19). MAbs have high potential as therapeutics and prophylactics against viral infections due to their high specificity and their ability to enhance immune responses. Currently, more than 20 mAbs against SARS-CoV-2 spike protein entered clinical development [3,4]. However, their efficacy as a single agent or combined in a cocktail is threatened by the emergence of escape variants [5,6,7,8].

Protein engineering platforms that integrate a synthetic compound library and efficient in vitro selection strategies, such as ribosomal, phage or yeast displays, open avenues for the development of novel therapeutics. Suitable drug candidates are identified by screening of engineered proteins whose backbone structure is maintained intact while varying a few key amino acids located on the protein surface to create a binding interface. Prominent examples are the heavy-chain variable domain of camelid antibodies, also known as nanobodies, which are characterized by small size (14 kDa), high stability (Tm up to 90 °C) and can be assembled in multivalent proteins. Recently, neutralizing nanobodies against SARS-CoV-2 have been successfully generated using this approach [9,10]. Designed Ankyrin Repeat Proteins (DARPins) offer another type of high-affinity binders that can be selected by in vitro selection techniques without immunization. DARPins are engineered proteins, whose scaffold is derived from naturally occurring ankyrin repeats. Plückthun and colleagues at the University of Zürich, Switzerland, initially conceived the strategy and established the methods to create new molecules based on a consensus repeat module that assembles in an array of variable lengths displaying a concave binding surface [11,12]. The team that participated in the elaboration of this platform then developed clinical-stage applications and commercialization of the technology in the field of medical therapy [13,14]. In this review, we highlight the main structural and biophysical properties of engineered DARPins for viral indications and discuss their potential for developing novel antiviral strategies.

## 2. Ankyrin Repeat Containing Protein

**Figure 1:**
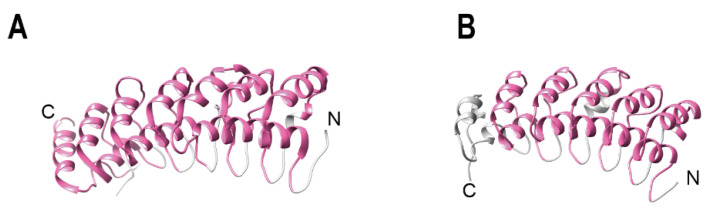
Examples of naturally occurring ankyrin repeat containing proteins. (**A**) Ankyrin repeat containing protein p100/NF-κB2 (Q00653). Structure of the C-terminal domain (PDB 4OT9, residues 483-756). The ankyrin repeats are colored in pink. (**B**) Ankyrin repeat containing protein IκBα (P25963). Structure of IκBα in complex with NF-κB (PDB 1NFI, residues 70-282). The ankyrin repeats domains are colored in pink.

Ankyrin repeats were discovered in 1987 in the yeast cell-cycle regulators Swi6 and cdc10 and the *Drosophila melanogaster* developmental regulator Notch [15]. In the same study, a related 33 amino acid repeat was found in the developmental regulator Lin-12 of the nematode *Caenorhabditis elegans* [15]. Subsequently, the same motif was found in other molecules and named after the human cytoskeletal protein ankyrin, which contains 24 repeats of this motif [16]. Ankyrin repeats are present in a large variety of proteins, including transcriptional regulators, developmental regulators, and cellular cytoskeletal organizers. Ankyrin repeat proteins are one of the most abundant repeat protein families, and the number of repeats found in a single protein may vary considerably [17,18,19,20,21,22]. Notably, 26% of ankyrin repeat proteins contain multiple arrays, with the upper limit of the size of an array of repeats being about 120 copies [17]. Yet, the arrays generally consist of four to six repeats [17,20]. About 85% of the arrays belong to eukaryotic proteomes, 13.0% to bacteria, 1.4% to viruses and 0.1% to archaea [17]. Ankyrin repeat containing proteins mediate protein–protein interactions, thus are fundamental for many cellular processes, such as transcriptional regulation, innate immunity, signal transduction and cell cycle control [20,22]. As a notable example, ankyrin repeat proteins have key functions in the signaling pathway mediated by NF-κB, being present in the NF-κB precursor p100 [23], and in the Iκ-B inhibitor [24,25] (Figure 1A,B). The repeats are required for the cytoplasmic retention of the transcription factors. Interestingly, a conformational change has been demonstrated for one or more ankyrin repeats of IκBα, which are disordered in solution, but acquire the ankyrin fold when bound to the target protein [26,27].

**Figure 2:**
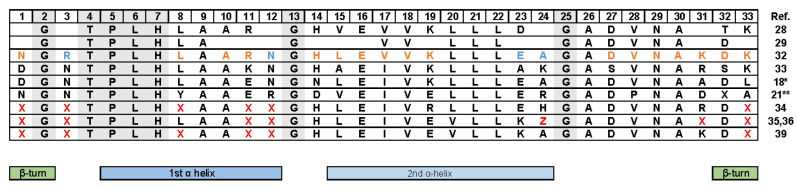
Ankyrin repeat consensus sequence. Letter refer to amino acids (1 letter code). Residue are numbered according to the UniProt annotation of the ankyrin repeat motif. In black: conserved residue. In blue: non-conserved residue. In orange: semi-conserved residue. X = any amino acid, except C, G, and P. Z = H, N, or Y. * bacterial sequence consensus. ** archea sequence consensus. The amino acids that are structurally important are highlighted in grey [18,21,28,29,32,33,34,35,36,39].

**Figure 3:**
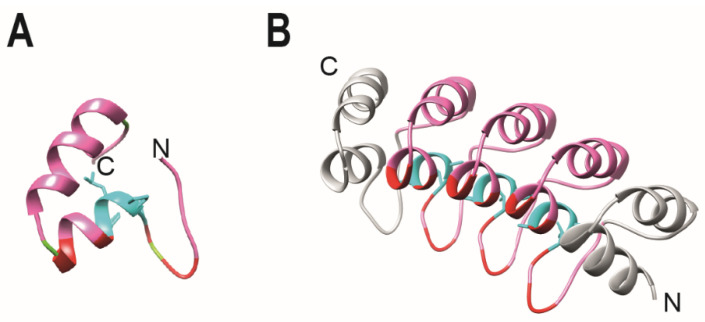
Structure of a monoDARPin domain. (**A**) Library module. The library module is colored in pink, with the conserved TPLH residues in cyan. Residues then can be randomized are indicated in red. (**B**) Homology model of monovalent DARPin R2 generated by SWISS MODEL (SIB) using the consensus designed ankyrin repeat domain PDB ID 2xee and the sequence of mono DARPin R2 [39]. The N-cap and C-cap are colored in grey.

The structural fold that forms a linear solenoid with a continuous hydrophobic core and a large solvent-accessible surface defines ankyrin repeats. Each repeat exhibits a conformation consisting in a β-hairpin and two antiparallel α-helices. Finally, the multiple consecutive repeats stack together, forming a scaffold that presents highly specific binding surfaces through which protein–protein interactions are mediated. Comprehensive reviews describe in more detail the structural features and biophysical properties of the ankyrin repeats [18,19,21,22,28,29,30,31]. Multiple sequence alignments of natural ankyrin repeat proteins and crystallographic analysis revealed a consistent pattern of key residues that preserve the structural integrity of the motif. Residues T4, P5 and H7 dictate the structural fold that forms a linear solenoid (Figure 2 and Figure 3A). Moreover, three glycine residues are conserved at consensus positions 2, 13 and 25. G2 terminates the β turn, whereas G13 and G25 terminate the repeat helices [18,20,21,28,29,32,33,34,35,36]. The high number of repeats found in nature allowed the design of robust consensus sequences [34,37]. A key feature is that additions or substitutions within the primary sequence of ankyrin repeats are tolerated in the loop regions that are less conserved, flexibility that provides the basis for the recognition of a high diversity of binding partners [20,38]. This property was exploited to generate combinatorial libraries [34,36].

## 3. Design Ankyrin Repeat Proteins (DARPins)

The consensus sequence of the ankyrin repeat opened the way to the design of engineered proteins. The structure and biochemical analysis of the two first consensus-designed ankyrin repeat proteins revealed that the constructs were well expressed in *E. coli*, correctly folded, and were thermodynamically more stable than their natural counterparts [32,35]. Thus, the consensus amino acid sequence contained all information required to define the repeat fold, and the designed repeats were self-compatibles and could stack together to present a compact groove-like binding surface.

These first generic proteins served as prototypes for engineering proteins with novel binding specificities. A technical breakthrough consisted in the generation of combinatorial libraries based on the consensus sequence and the development of efficient in vitro selection procedures for isolating molecules able to bind tightly to the ligand of choice [12,34,40,41]. The consensus repeat sequence comprises structurally important residues, and non-conserved residues that allowed the determination of six positions (represented as X in Figure 2) that were randomized in a library format with any amino acid except cysteine, to eliminate disulfide formation, and glycine and proline, which are structurally unfavorable. Library modules consisting of either two or three internal repeats were inserted between designed N- and C-terminal capping ankyrin repeats that shield the hydrophobic core from the solvent. Initially derived from the human guanine-adenine-binding protein [40], the design of the cap structures have been improved over the years, based on experimental observations of their importance also for overall folding and stability [12,38,40,42,43]. Moreover, based on the finding that both N- and C-terminal capping repeats can be involved in target binding, capping residues were subsequently randomized to further increase the variability of a library with two internal repeats [42,43]. The theoretical diversity for libraries comprising DARPins with three internal repeats is up to 3.8 × 10^23^ molecules [40]. The resulting DARPins with two internal and three internal repeats are 124 and 157 amino acids in size, respectively, and consists of a small (14–17 kDa) α-helical scaffold, which is highly stable and has a low tendency to aggregate (Figure 3).

**Figure 4:**
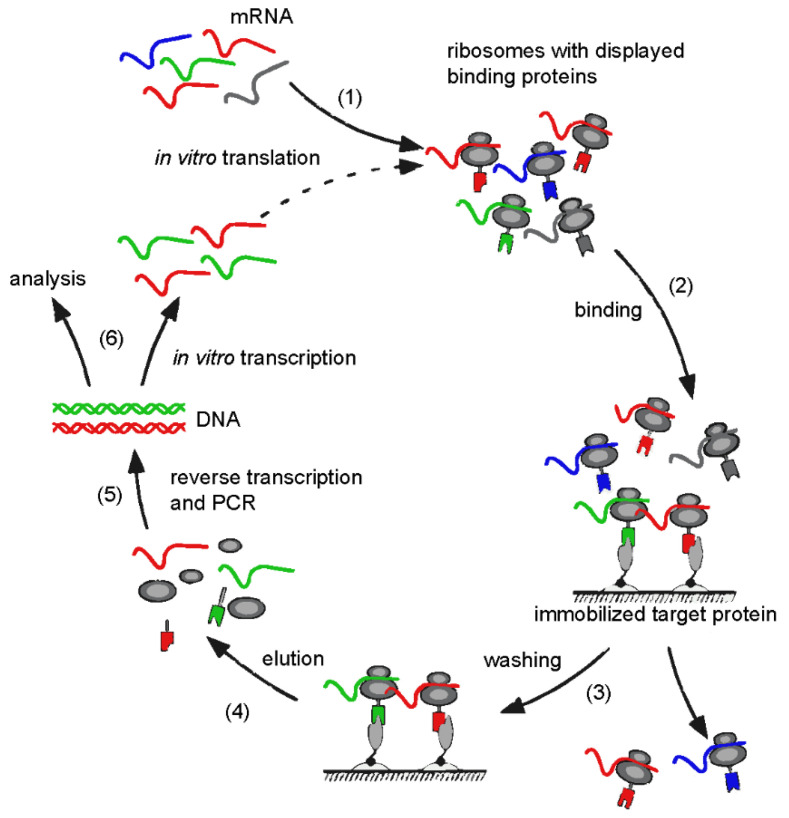
Schematic representation of a ribosome display selection cycle. An mRNA library encoding the proteins of interest without stop codon is translated in vitro (1). After cooling, the translation yields stable ternary complexes of mRNA, ribosomes and nascent polypeptides. These complexes are used for the binding selection on the immobilized target (2). After binding of the polypeptides to the target protein, unbound complexes are washed away (3). The mRNA of the bound complex is eluted by dissociating the ribosomal complex with EDTA (4). A reverse transcription reaction followed by PCR yields the genetic information of the selected clones (5). The amplified genes can then used as input for the next selection round starting with in vitro transcription (6) or cloned intro plasmids for analysis. (adapted from [56]).

From such libraries, DARPins with high affinities towards almost any protein target can be generated, using predominantly ribosome display as a selection method [11,36,44]. Other strategies include phage display [45,46] or yeast display [47]. However, selection technologies that involve microbial cells exhibit a major drawback in terms of diversity, as the number of molecules reached in practice after transformation strongly depends on the host system. Ribosomal display is an in vitro selection method based on stalled ribosomal complexes, linking the nascent protein phenotype (e.g., binding to a target protein) with its corresponding mRNA genotype during the selection process (Figure 4). For this purpose, the library of interest is transcribed into mRNA and translated in vitro with all *E.coli* features necessary for efficient translation under the control of a T7 promoter. The elimination of the translational stop codons on the mRNA ensures that the newly synthesized peptide and its encoding mRNA fail to be released from the ribosome. The ribosome-mRNA-protein complex is subsequently used for affinity selection on an immobilized or soluble target. After washing steps to discard the majority of non-binders, the retained mRNAs of complexes displaying binding-competent DARPins are recovered by reverse-transcription (RT) and polymerase chain reaction (PCR) to yield the sequences encoding the target-specific binders. The resulting PCR products are used for the next cycle of ribosome display selection or cloned into expression vectors and analyzed. As the ribosomal display translational machinery is derived from *E. coli*, in which the DARPin scaffolds are well expressed and folded, and high physical diversities (up to 10^12^ molecules) can be obtained [36]. Furthermore, the PCR step can be combined with random mutagenesis enabling affinity maturation and directed in vitro evolution [48,49]. Highly specific DARPins with subnanomolar binding affinities to a wide range of proteins including kinases and growth factors have been generated using this methodology [41,48,50,51]. DARPins are advantageous compared to some other naturally occurring proteins for biotechnological and medical applications because of the lack of disulfide bridges and post-translational modifications that enable fast, low-cost, large-scale microbial production. Subsequent conjugation to effector molecules such as radio-ligands, toxins and fluorophores is possible by site-specific insertion of cysteine residues [36]. Finally, the high thermostability may overcome cold-chain requirements for storage.

The immunogenicity of proteins and associated in vivo development of antibodies may diminish the bioactivity and effectiveness of the therapeutics [52]. The scaffold of DARPins is not prone to aggregate and derives from ankyrin repeats, which are present in the natural proteins of humans and other species. Despite other factors, which may influence the tolerability of proteins, low aggregation propensity and humanization of antibodies could be shown to positively influence the immunogenic potential of a therapeutic protein [53,54]. An example of the immunogenic potential of DARPins was presented in a first-in-human study of MP0250, a DARPin drug candidate inhibiting vascular endothelial growth factor (VEGF) and hepatocyte growth factor (HGF) showed sustained exposure during the trial, indicating the absence of clearing antibodies, despite some levels of anti-drug antibodies in some patients [55].

## 4. Viral Applications

The DARPin technology may cover many applications in biomedical research, such as diagnostics, infectious disease therapy or 5 mmune-oncology [11,13]. Originally, DARPins were used as effective tools for cell-type specific gene delivery of viral vectors and tumor killing. Several approaches have been explored using lentiviruses, adeno-associated viral (AAV) vectors, adenovirus serotype 5 (Ad5), and oncolytic recombinant measles virus [11,57]. DARPins can either target viral proteins, cellular components or both (Table 1). For example, tumor cells have been targeted using bispecific molecules comprising a DARPin binding to Ad5, thereby blocking the natural receptor binding site of the virus, and a high affinity DARPin targeting human epidermal growth factor receptor 2 (Her2), a marker of aggressive tumors [51,58]. Oncolytic recombinant measles virus was retargeted by elongating the measles virus attachment protein with Her2/neu-specific, epithelial adhesion molecule (EpCAM)-specific or epidermal growth factor receptor (EGFR)-specific DARPins [59,60]. DARPins have been used to modify the tropism of lentiviral vectors and AAV vectors, two widely used gene transfer systems [57]. Lentiviral vectors pseudotyped with engineered measles virus glycoproteins (hemagglutinin H and fusion F protein) have been retargeted to CD340/Her2/neu positive breast cancer cells [61], and to CD4 positive T lymphocytes [62]. Finally, AAV vectors have been retargeted to breast cancer cells by using the surface protein Her2/neu by fusing the DARPin domain to the VP2 protein on the viral capsid [63,64].

A way to circumvent viral Infection is to target cellular components or processes essential for the virus infectivity or pathogenicity. DARPins can also act intracellularly upon ectopic expression in mammalian cells. A major advantage of DARPins being their solubility and stability even in the case of the reducing conditions of the intracellular environment where they can block critical steps of the virus life cycle such as viral replication, assembly and egress. Such an experimental approach was followed by targeting the zinc finger domain of the cellular deacetylase HDAC6, thereby blocking the interaction with ubiquitin, interfering with viral uncoating mechanisms and impairing infection by Influenza and Zika virus [65]. Another antiviral strategy is to target a viral protein that plays a key role in the viral cycle. SupT1 cells that stably express an artificial ankyrin repeat molecule (AnkGAG1D4) binding to the human immunodeficiency virus (HIV) Gag polyprotein showed a reduced permissiveness to HIV-1 infection [34]. Subsequent studies investigated the molecular mechanisms whereby AnkGAG1D4 inhibited HIV replication, showing that the intracellular expression of AnkGAG1D4 affected the late stages of virus production [66,67,68,69]. Despite this as a promising strategy, intracellular DARPin delivery may limit in vivo administration of the molecule. As an alternative, antiviral drug development using large molecules may focus on extracellular components. Such a design has been chosen for human HIV, by targeting CD4 [70]. The interaction between the envelope glycoproteins and the cellular receptor CD4 and a coreceptor (typically CCR5 or CXCR4) initiates HIV infection. Selected DARPins specific for human CD4 blocked HIV entry and potently inhibited infection of genetically divergent HIV isolates with IC50 values in the low nanomolar range [70]. 

Direct blocking of viral entry by neutralization is a classical approach, as it allows stopping the pathogen before it can take control of the host cell’s machinery for multiplication and dissemination. The first proof-of-concept that the DARPin technology can neutralize viruses by targeting a viral component was given by a study on Lactococcal Phage TP901-1 [71]. Phage infection is initiated by binding the phage receptor binding protein located within the baseplate at the distal part of the tail, to its receptor on the host cell surface. DARPins directed against the hetero-oligomeric macromolecular multiprotein complex, which comprises part of the baseplate of the phage could inhibit TP901-1 infection. Furthermore, the DARPin technology was employed to design HIV inhibitors targeting the envelope glycoprotein gp120 [72,73]. Broad neutralization could be achieved by a DARPin molecule directed to the V3 crown of HIV envelope glycoprotein gp120, providing relevant information for the development of novel antiviral strategies against HIV.

**Table 1:** Viral applications.

DARPin Name	Target/Epitope	Mechanism of Action	Application	Ref.
1D3 2E6	Ad5 knob Her2	Bispecific adapter to retarget Ad5 to tumor cells	Tumor retargeting of Ad5	[51,58]
9.26 9.01 9.16 G3 9.29 H14R	Her2	Engineered MeV attachment protein Hemagglutinin unable to bind to its natural receptor genetically fused to a Her2-specific DARPin binding domain	Tumor retargeting of pseudotyped lentiviral vector	[61]
D29.2	CD4	Engineered MeV attachment protein Hemagglutinin unable to bind to its natural receptor genetically fused to a CD4-specific DARPin binding domain	Retargeting pseudotyped lentiviral vector to CD4^+^ cells	[62]
9.16 9.29 9.26 G3	Her2	Engineered MeV attachment protein Hemagglutinin unable to bind to its natural receptor genetically fused to either Her2, EpCAM or EGFR-specific DARPin bindingdomain (Monospecific or bispecific G3 +EC4)	Tumor retargeting of recombinant MeV	[59]
C9 EC4	EpCAM			
E.01 E.68 E.69	EGFR			
E.01	EGFR	Engineered MeV fusion protein (F) that can be activated by tumor-associated matrix metalloprotease, genetically fused to EGFR-specific DARPin binding domain	Tumor retargeting of recombinant MeV	[60]
9.29	Her2	Engineered capsid protein VP2 fused to Her2-specific DARPin binding domain and ablated natural receptor binding site on VP3	Tumor retargeting of AAV	[63,64]
EC1	EpCAM	Engineered capsid protein VP2 fused to EpCAM-specific DARPin binding domain and ablated natural receptor binding site on VP3	Tumor retargeting of AAV	[64]
D55.2	CD4	Engineered capsid protein VP2 fused to CD4-specific DARPin binding domain and ablated natural receptor binding site on VP3	Retargeting AAV to CD4^+^ cells	[64]
E.01	EGFR	Engineered capsid protein VP2 fused to human FK-binding protein (FKBP) and ablated natural receptor binding site on VP3. Adapter protein that consist of a modified FKBP rapamycin binding domain of mTOR fused to mCherry and EGFP-specific DARPin binding domain	Adaptor for the tumor retargeting of AAV	[74]
E.01	EGFR	Engineered capsid protein VP2 fused to EGFR-specific DARPin binding domain and ablated natural receptor binding site on VP3	Tumor retargeting of AAV	[75]
F10	Human HDAC6 zinc finger domain	Impairs interaction with ubiquitin and infection by influenza and Zika virus	antiviral	[65]
Ank^GAG^1D4	HIV-1 Gag precursor	Interferes with late stages of HIV-1 capsid assembly	antiviral	[34,66,67,68,69]
D1.1-D6.1 D23.2 D25.2 D27.2 D29.2 D55.2 D57.2	CD4	Inhibit HIV-1 cell entry by blocking the binding to CD4, the main receptor of HIV-1	antiviral	[70]
D_18 D_19 D_20	Lactococcal phage TP901-1 BppU BppL complex	Neutralization of phage TP901-1 by blocking receptor binding	antiviral	[71]
bnD_1 bnD_2 bnD_3	V3 crown of the HIV-1 envelope protein (gp120)	Neutralization of HIV-1 by blocking receptor binding	antiviral	[73]
5m3_D12	V3 loop of the HIV-1 envelope protein (gp120)	Neutralization of HIV-1 by blocking receptor binding	antiviral	[72]
R1 R2 R3	RBD domain of SARS-CoV-2 spike	Neutralization of SARS-CoV-2 by blocking receptor binding	antiviral Therapeutic treatment of COVID-19	[39]

Abbreviations: AAV, Adeno associated virus; Ad5, Adenovirus serotype 5; COVID-19, corona virus disease 2019; DARPin, designed ankyrin repeat protein; EGFR, epidermal growth factor receptor; EpCAM, Epithelial cell adhesion molecule; HIV-1, human immunodeficiency virus type 1; MeV, measles virus; SARS-CoV-2, severe acute respiratory syndrome coronavirus type 2.

## 5. Ensovibep

Ensovibep, a multispecific therapeutic developed for the treatment of COVID-19 is the first DARPin-based antiviral evaluated in clinical trials [39,76]. Clinical stage developments of DARPins have been previously demonstrated for several disease models, among them cancer indications [77] and neovascular age-related macular degeneration [78]. The COVID-19 pandemic has created urgent needs for innovative therapeutics that could be available on a global scale to control the spread of the virus. The rapid development of ensovibep demonstrates the effectiveness of the DARPin technology against an emerging virus.

The SARS-CoV-2 spike (S) glycoprotein is the target of neutralizing antibodies. The S glycoprotein is a transmembrane trimer with two functional subunits: S1, comprising the N-terminal domain (NTD) and the receptor-binding domain (RBD) responsible for interaction with the angiotensin-converting enzyme 2 (ACE2) host receptor, and S2, that mediates fusion of the viral and cellular membranes [79,80,81,82,83,84,85]. All monoclonal antibodies for COVID-19 are neutralizing, and the majority of them prevent SARS-CoV-2 entry by blocking engagement with ACE2 by targeting epitopes within the RBD [3,4]. Notably, mAbs developed to treat COVID-19 are the first mAbs authorized for use in a therapeutic setting for a respiratory pathogen, proving the concept that virus neutralization could be an effective antiviral strategy, at least when administered at early stages of the disease [3,4].

**Figure 5:**
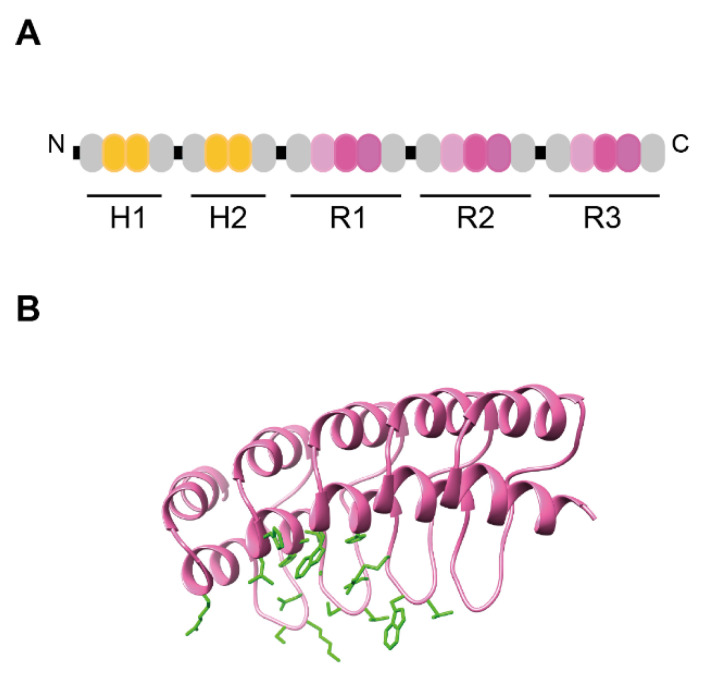
Schematic representation of ensovibep and structure of monoDARPin R2. (**A**) Schematic representation of ensovibep. The protein comprises two HSA-binding DARPin domains for in vivo half-life extension and three DARPin domains that bind the RBD of the SARS-CoV-2 spike trimer. The HSA-binding DARPins contain two internal repeats (indicated in yellow) and the RBD binding DARPins three internal repeats (indicated in pink). The N-Cap and C-Cap repeats are represented in grey. The linker sequences are colored in black. (**B**) Binding surface of monovalent DARPin R2. Homology model of monovalent DARPin R2 generated by SWISS MODEL (SIB) using the consensus designed ankyrin repeat domain PDB ID 2xee and the sequence of DARPin R2 [39]. The library modules are colored in pink. The side chains of the residues interacting with the RBD of the SARS-CoV-2 spike trimer, as defined by cryo-EM analysis are highlighted in green.

Ensovibep is structurally different from antibodies, and consists of a single chain of five DARPin binding domains, which are linked together, comprising two human serum albumin (HSA)-binding DARPin domains for in vivo half-life extension and three DARPin domains that bind the RBD of the SARS-CoV-2 spike trimer (Table 1) (Figure 5A,B) [39].

The systemic half-life greatly influences the therapeutic efficacy of proteins. Small proteins are quickly eliminated due to renal clearance, and liver metabolism. Several techniques are available to extend their half-life, such as PEGylation and fusion to human serum albumin [86]. Multispecific DARPins containing HSA-binding domains have improved pharmacokinetic properties and a serum half-life of approximately 2 weeks in humans [87]. DARPin domains can be linked to each other without any negative effect on folding, expression or biological function. Ensovibep has two HSA-binders and a two-week half-life [39],67].

Ensovibep recognizes SARS-CoV-2 with high affinity and specificity. The three RBD-binding domains are sequence related and target a common epitope but they are not identical in their paratopes. This design allows the protein to bind with high avidity and may cover a potency loss if one domain is susceptible to a mutation in the binding site. Indeed, the mutational analysis revealed that a single ensovibep molecule binds the trimeric S glycoprotein in a cooperative manner to improve potency [39]. Additional structural analysis showed that the three DARPin domains could target simultaneously the receptor-binding ridge on each RBD of the spike trimer, locking the spike in an open conformation and occluding the ACE2-binding site [39]. Ensovibep met its clinical endpoints in the EMPATHY study, showing significantly reduced viral loads compared to placebo and patients treated with ensovibep recovered faster [88]. Ensovibep was safe and well tolerated. 

## 6. Concluding Remarks

Protein–protein interactions regulate essential cellular processes. Derived from natural ankyrin containing proteins, DARPins provide a binding scaffold with exceptional structural and biophysical properties, such as a rigid core, high thermostability, easy assembly of multiple binding domains and large scale microbial production. Several DARPin domains can be linked to each other without major impact on folding, expression or their stability. This makes them ideal building blocks for designing structures that are more complex. The multispecific design of DARPin therapeutics confers a key advantage in the context of the emergence of virus escape mutants. As monoclonal antibodies, DARPins may be susceptible to amino acid changes in the target sequence. However, the modular architecture of the molecule enables rapid replacement of a domain with a newly selected one when mutations arise on the viral target. This feature is particularly important, as RNA viruses possess a high mutation rate and enormous adaptive capacity. 

DARPins have been successfully generated neutralizing Phage TP901-1, HIV and SARS-CoV2 by blocking the interactions between the viruses and their cognate receptors, thereby inhibiting cell entry and infection. Enveloped RNA viruses, with their high potential for mutations and epidemic spread, are among the most common source of emerging human pathogens, and most are of zoonotic origin [89,90,91]. Host cell attachment and entry represent major barriers for cross-species transmission and are decisive steps for host-range, tissue tropism, and disease potential of the virus. Some viruses, among them SARS-CoV-2, evolved glycoproteins that bind specifically and with high affinity to cell surface receptors. However, emerging viruses have a priori no selection pressure to evolve the capacity to recognize human receptors since virus–host co-evolution is driven mainly by long-term relationships with the reservoir hosts. Many viruses use much broader mechanisms to invade the host cells, such as binding to glycan-binding proteins via glycans on the viral glycoprotein or binding to host cell phosphatidylserine receptors via the lipids of the viral envelope [92,93,94].

First demonstrated by Mercer and Helenius for the Vaccinia virus [95], some viruses mimic apoptotic bodies that can be engulfed by cells through clearance mechanisms. Apoptotic mimicry is now recognized as a major entry strategy used by a broad spectrum of viruses, including important emerging pathogens, such as Ebola, Dengue, West Nile, Zika, Hantaan and Andes viruses [96,97,98,99,100,101,102,103]. These viruses acquire phosphatidylserine in their envelope during budding. The cellular phosphatidylserine receptors, which mediate their entry are molecules of the T-cell immunoglobulin and mucin (TIM) receptor family, in particular TIM-1 and TIM-4, and receptor tyrosine kinases of the Tyro3/Axl/Mer (TAM) family [92,94]. Thus, these viruses can exploit highly conserved clearance and immunomodulatory processes developed by the eukaryotic cells to easily cross the species barrier. Targeting viral entry is a promising approach because it neutralizes the pathogen before it can take control of the host cell’s machinery for multiplication and dissemination. However, additional strategies than interfering with receptor binding are needed to combat viruses that invade cells using less selective mechanisms of entry or multiple internalization pathways. The identification of additional sites of vulnerability on viruses is a clear priority for the development of efficacious antiviral strategies. A common, but essential process for all enveloped viruses is the fusion between virus and cell membranes [104,105]. An appealing approach is to target proteins that are involved in the fusion process, including the viral fusion proteins themselves. To date, fusion inhibitors include small molecules, peptides, nanobodies and monoclonal antibodies [47,106,107,108,109]. The modular architecture of DARPins offers new possibilities of design and opens an area of antiviral research that will certainly prove fruitful in the future. 

## Data Availability

Not applicable.
